# Mitosis in Neurons: Roughex and APC/C Maintain Cell Cycle Exit to Prevent Cytokinetic and Axonal Defects in *Drosophila* Photoreceptor Neurons

**DOI:** 10.1371/journal.pgen.1003049

**Published:** 2012-11-29

**Authors:** Robert Ruggiero, Abhijit Kale, Barbara Thomas, Nicholas E. Baker

**Affiliations:** 1 Department of Genetics, Albert Einstein College of Medicine, Bronx, New York, United States of America; 2 Laboratory of Biochemistry, National Cancer Institute, National Institutes of Health, Bethesda, Maryland, United States of America; 3 Department of Developmental and Molecular Biology, Albert Einstein College of Medicine, Bronx, New York, United States of America; 4 Department of Ophthalmology and Visual Sciences, Albert Einstein College of Medicine, Bronx, New York, United States of America; New York University, United States of America

## Abstract

The mechanisms of cell cycle exit by neurons remain poorly understood. Through genetic and developmental analysis of *Drosophila* eye development, we found that the cyclin-dependent kinase-inhibitor Roughex maintains G1 cell cycle exit during differentiation of the R8 class of photoreceptor neurons. The *roughex* mutant neurons re-enter the mitotic cell cycle and progress without executing cytokinesis, unlike non-neuronal cells in the *roughex* mutant that perform complete cell divisions. After mitosis, the binucleated R8 neurons usually transport one daughter nucleus away from the cell body into the developing axon towards the brain in a kinesin-dependent manner resembling anterograde axonal trafficking. Similar cell cycle and photoreceptor neuron defects occurred in mutants for components of the Anaphase Promoting Complex/Cyclosome. These findings indicate a neuron-specific defect in cytokinesis and demonstrate a critical role for mitotic cyclin downregulation both to maintain cell cycle exit during neuronal differentiation and to prevent axonal defects following failed cytokinesis.

## Author Summary

Neurons generally differentiate and never divide again. One barrier to understanding the mechanisms has been the paucity of genetic mutations that result in neuronal cell cycles. Here we show that mutation in three genes lead to cell cycle re-entry by a particular class of developing photoreceptor neurons in the fly retina. Strikingly, these neurons do not complete cell division but only divide their nuclei. The binucleated neurons then typically retain one nucleus in its normal location in the cell body, while transporting the other into the growing axon like other axonal material. Our findings identify Cyclin A regulation as crucial to maintaining cell cycle exit by at least some neurons and identify a neuron-specific defect in cell division as a further barrier to neuron proliferation. Because defects in transporting axonal material have been implicated in the origin of multiple neurodegenerative diseases, our findings also suggest a possible connection between defective cell cycle regulation and neuronal cell death.

## Introduction

The near universality of neuronal cell cycle exit implies robust barriers to division. These could include additional neuron-specific and redundant barriers to cell cycle entry and progression, and surveillance mechanisms to eliminate neurons that evade them. Even in tumors, to date the only candidate for a dividing neuron is the horizontal interneuron suggested to be the cell of origin for retinoblastoma [1].

It is often speculated that the cell cycle might be lethal for neurons, or that inability to duplicate axons may prevent neuronal division. In the case of horizontal cells that give rise to retinoblastoma in the mouse retina, mutations at three Rb-family loci are required for transformation [2]. Such genotypes cause other types of retinal neuron to die [2]. In *Drosophila*, in which *rbf1* is the only positively-acting Rb family gene, differentiating retinal neurons only re-enter the cell cycle if both Rbf1 and the p21/27-like gene *dacapo* are removed, and the contribution of mitotic *rbf dap* neurons to the differentiated nervous system has not been determined [3]. There are reports of cell cycle entry by a subset of neurons in the normal mammalian CNS, including retinal ganglion cells, but it is thought that such cell cycles are usually not completed [4]. It has been suggested that unscheduled cell cycle re-entry contributes to neurological diseases [5]–[9]. On the other hand, substantial evidence links multiple neurodegenerative conditions to defects in axonal trafficking [10]–[13]. Overall, much remains to be learned concerning cell cycle status and mechanisms in neurons, and its relationship to disease.

**Figure 1:**
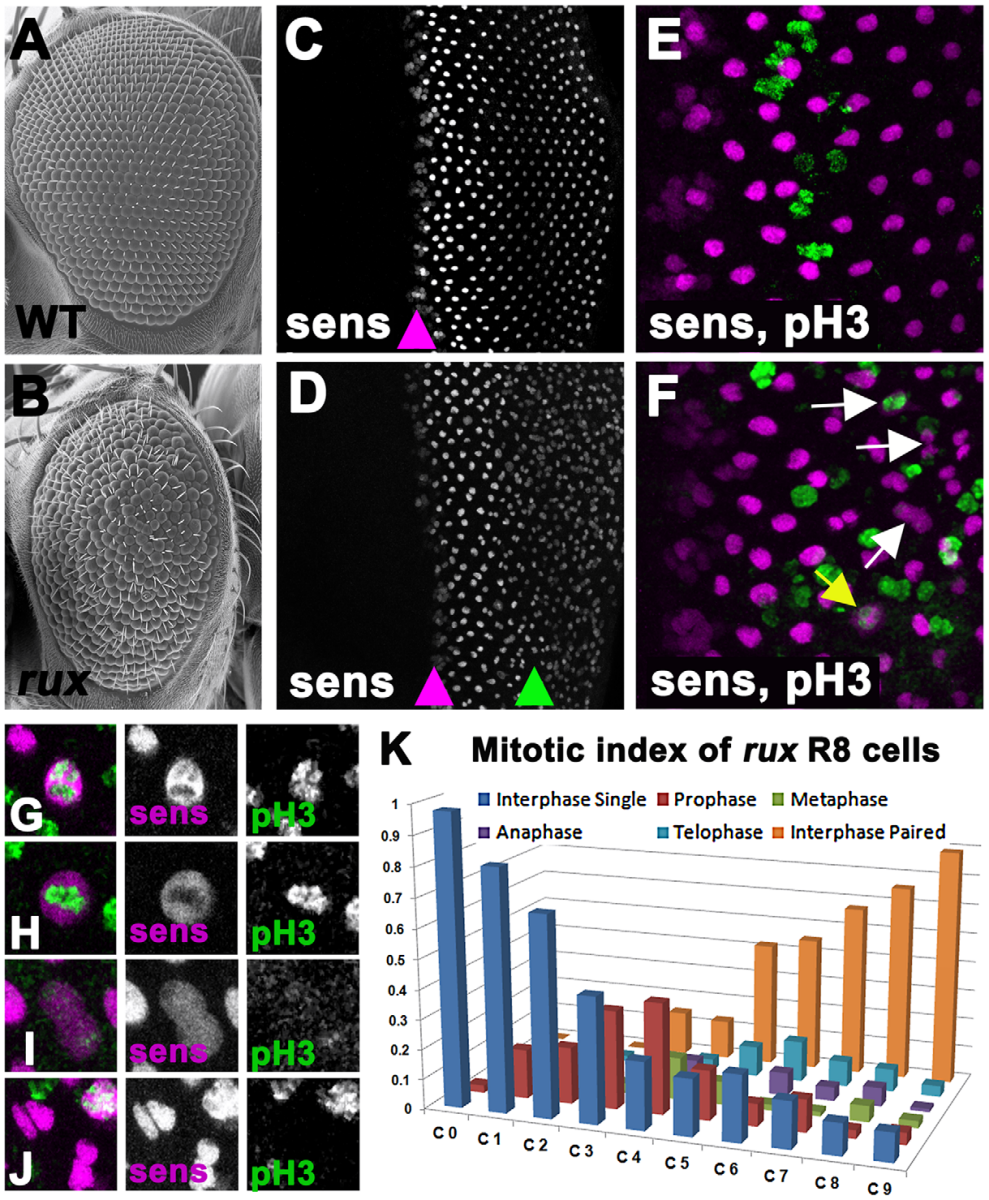
Mitotic R8 cells re-enter the cell cycle in *rux* mutants. Scanning electron micrographs of the wild type (A) and *rux* (B) adult eyes. In both wild type (C) and *rux* (D) third instar eye discs, normal Sens staining appears in intermediate group cells (purple arrowheads) before becoming isolated to single R8 cells. In *rux* R8 nuclei become disorganized after their number increases (D, green arrowhead). Mitotic figures labeled with H3p (green) are seen only in non-neuronal cells during the Second Mitotic Wave of wild type eye discs (E), but in *rux* discs (F) mitotic R8 cells and paired R8 nuclei are also seen (yellow and white arrows). G–K. Mitotic R8 cells from a *rux* eye disc in prophase (G), metaphase (H), anaphase (I), and as closely paired nuclei (J). K. Mitotic index of R8 cells showing the fraction of mitotic R8 cells in each of the first 10 columns (n = 5 eye discs and 620 R8 cells). See also Figure S1.

In this paper, we describe cell cycle re-entry in differentiating photoreceptor neurons lacking the cyclin dependent kinase inhibitor, *roughex*, or certain components of the Anaphase Promoting Complex/Cyclosome (APC/C), and the cellular consequences of such deregulated neuronal cell cycles in the differentiating *Drosophila* eye. The *Drosophila* adult eye is derived from the eye imaginal disc, which during the last larval stage is transformed from a sheet of undifferentiated, proliferating cells to highly organized neuroepithelium (Figure 1) [14], [15]. The recruitment and specification of cells begins at the posterior of the organ and progresses anteriorly as diffusible signals from more differentiated cells induce undifferentiated cells to exit the cell cycle in G1 [16]. These G1 arrested cells change shape to form a transient depression called the morphogenetic furrow that approximately separates the progenitor cell population from the region where retinal patterning and differentiation has begun (Figure 1) [15]. An array of R8 photoreceptor neuron precursor cells is selected from clusters of cells called intermediate groups that express the transcription factors Atonal and Senseless [17]–[20]. Cell interactions refine the expression of Atonal and Senseless to individual cells in each intermediate group that are specified as R8 photoreceptor precursors, the first neuron of each unit eye or ommatidium [21], [22]. R8 cells then coordinate the recruitment of some neighboring cells to the ommatidium while the remaining cells undergo an additional round of cell division, called the Second Mitotic Wave, to generate more progenitors required to form a complete unit eye, or ommatidium (Figure 1) [23], [15], [24], [16]. Soon after being specified, photoreceptor neurons form axons that grow below the basal surface of the disc and project to the optic lobe [23], [25], [26].

The roughex (rux) locus encodes a cyclin dependent kinase inhibitor that suppresses Cyclin A/CDK1 activity and regulates the cell cycle in several tissues [27]–[32]. Cyclin A is required for entry into mitosis in *Drosophila* whereas Cyclin E/CDK2 is required for entry into S-phase [33], [34]. Cyclin A is able to drive S phase entry if it is stabilized in G1, however, bypassing the requirement for Cyclin E [29], [35], [36]. Adult *rux* mutants exhibit deformed eyes composed of disorganized eye units containing irregular, reduced numbers of photoreceptors [28]. In *rux* mutant eye discs, G1 arrest is less effective both anterior and posterior to the morphogenetic furrow and generalized mitotic activity replaces the orderly second mitotic wave. The ectopic mitosis was previously thought to be limited to non-neuronal progenitor cells, and the fact that multiple R8 precursors were seen was attributed to an effect of proliferation on R8 specification [28]. Here, we report instead that cell cycle re-entry during R8 differentiation leads to the appearance of multiple R8 cells. The R8 neurons complete mitosis without undergoing cytokinesis, and their syncytial daughter nuclei become involved in axonal trafficking, demonstrating an unexpected link between the two main proposed mechanisms of neurodegeneration.

## Results

### Rux activity is required in R8 cells

Genetic mosaic analysis was used to assess the requirement for *rux* in distinct ommatidial cells. Adult *rux* mutant eyes have a reduced number of ommatidia that often lack a variable number of cells(Figure 1A, 1B) [28]. Mosaic eyes containing clones of the null mutation *rux^8^* were sectioned, and how often ommatidia developed normally despite containing *rux* null cells was determined for each cell type (Table 1). Strikingly, less than 1% of the mosaic, normal ommatidia contained mutant R8 cells. In addition, the *rux* mutation was tolerated in other photoreceptor cells less often than the 50% recovery that would be expected were it entirely neutral, consistent with a lesser requirement in cells other than R8. These findings indicate that the *rux* gene is directly required in R8 cells, and challenge the conclusion that R8 development is affected in *rux* as an indirect consequence of mitotic defects affecting other cells. It was unexpected that a cell cycle regulator would be required in R8 precursor cells, a post-mitotic neuronal cell type.

**Table 1:** Analysis of adult retina mosaic for *rux^8^*.

R cell	Number mutant	% mutant
R1	75	28%
R2	57	21%
R3	60	22%
R4	57	21%
R5	43	16%
R6	84	31%
R7	35	13%
R8	2	<1%

Shown is a summary of the *rux^8^* mutant photoreceptor cells recovered in mosaic ommatidia that were morphologically normal in adult sections. 270 morphologically normal, mosaic ommatidia were assessed in 44 eyes.

### Rux is required to maintain of cell cycle exit in R8 cells

The presence of extra R8 cells in *rux* mutants was observed previously using antibodies against the R8-specific apical membrane protein Boss and the pattern of expression of the R8-specific lac-Z reporter BB02 [28]. Thus R8 development must be abnormal by the time these markers are expressed, about 12 hours after specification. We re-examined R8 development using an antibody against the Senseless protein, which is expressed from the onset of R8 specification [37]. Senseless expression in wild type animals is normally seen in intermediate groups within the furrow and becomes isolated to a single R8 cell in the next column, column 0, thereafter being maintained in R8 cells for the remainder of eye development (Figure 1C). Contrary to the previous model, we found that intermediate group formation and the selection of a single R8 cell occurred almost normally in *rux* mutants, and that multiple R8 nuclei were not apparent until further posterior in the eye disc (Figure 1D). Furthermore, it was evident that these were associated with R8 cell mitosis.

Starting at column 3, Senseless labeling transiently expanded through the R8 cells, an appearance previously seen in mitotic photoreceptor neurons where the nuclear membrane breaks down [3]. Consistent with this, mitotic chromatin labeled by an antibody against Histone 3 phosphorylated on Ser 10 (H3p) [38] was seen in R8 cells from column 3–8 (Figure 1E, 1F), progressing through prophase, metaphase and anaphase morphologies, followed by Sens labeling of pairs of sens-positive R8 nuclei (Figure 1F–1J). The regular array of R8 nuclei also became less organized as their numbers increased. Figure 1K catalogs the mitotic stages of R8 nuclei in *rux*. They entered prophase between columns 0 and 3. Some quickly progressed through mitosis, since paired R8 nuclei in interphase were first seen in column 3, but R8 cells in anaphase and telophase were commonest between columns 7 and 10, and pairs of R8 nuclei accumulated most rapidly after column 6. Most or all R8 nuclei were paired by column 10. Taken together with the mosaic analysis, these data suggest defects in R8 number and patterning do not reflect defects in R8 specification but are a consequence of cell-cycle re-entry by R8 cells that have undergone fate specification.

Previous studies established that *rux* prevents division of non-neuronal cells by inhibiting CycA [30], [39]. The effect of reducing *cycA* gene dose on R8 cell cycles was examined to confirm CycA dependence. Consistent with this expectation, the number of and rate of R8 mitosis was significantly reduced in *rux^8^*;*cycA*/+ eye discs (Figure S1).

We also examined the mitotic status of other photoreceptor cell types. Based on quantifying the co-labeling of H3p with the pan-neuronal marker Elav, we estimate that only about 20% of photoreceptor cells other than R8 re-entered the cell cycle (data not shown).

### R8 cell mitosis in *rux* is insensitive to p21

**Figure 2:**
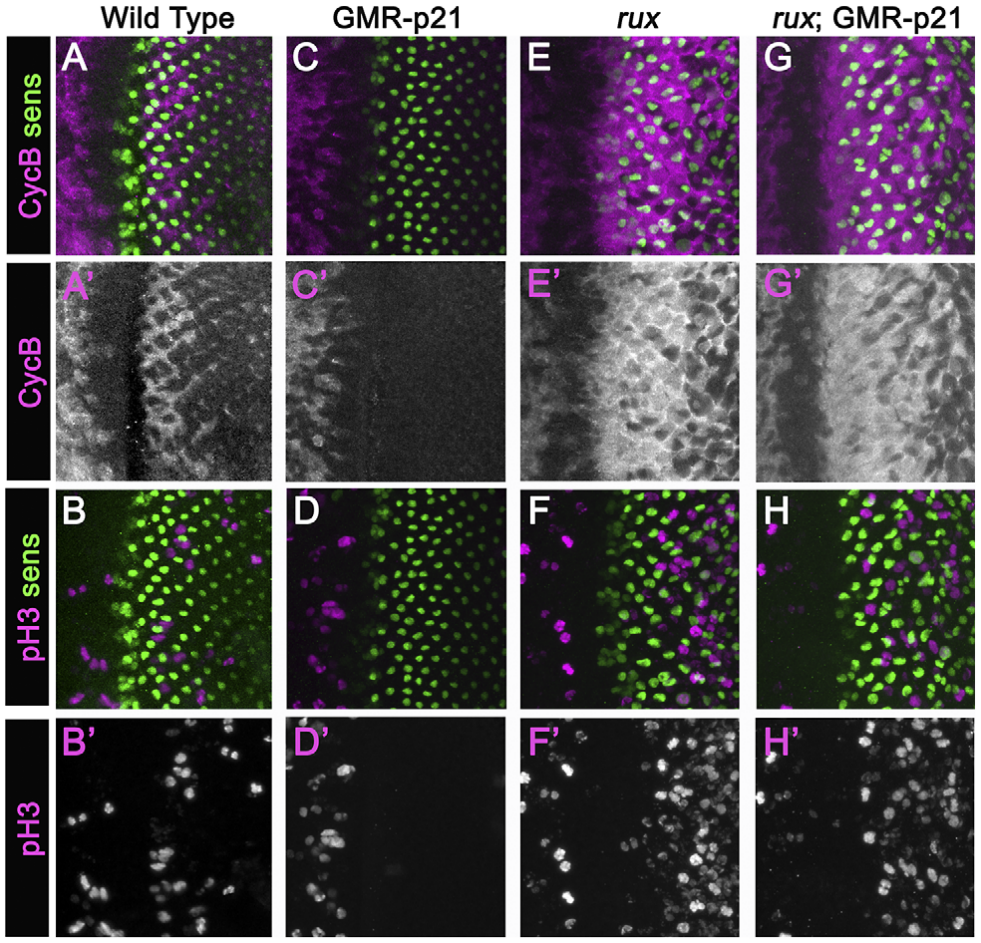
Mitotic activity in *rux* mutants is not affected by p21. Eye discs from different genotypes labeled with Sens (green) and Cyclin B (A,C,G,E) or H3p (B,D,F,H) in magenta. (A)(B) Wild type eye discs exhibit the pattern of cells entering the SMW. (C)(D) When p21 is expressed this blocks the Second Mitotic Wave. (E)(F) In *rux*, arrest ahead of the morphogenetic furrow is incomplete and truncated, and there is much more mitotic activity posterior to the furrow. (G,H). *rux*; GMR-p21 eye discs are identical to *rux* (compare panels E,F).

In an attempt to separate pre- and post-specification effects of *rux* we expressed human p21 using the GMR-p21 transgenic line [38]. This cyclin dependent kinase inhibitor (CDI) strongly inhibits G1 cyclin activity of CDK2 and CDK4 complexes, but is considerably less effective at inhibiting CDK1 [40]–[43]. Cell cycle progression was monitored by labeling for mitotic H3p and for Cyclin B, which accumulates in cells between S-phase entry and mitotic metaphase [44], [34](Figure 2A, 2B). GMR-p21 completely blocks the second mitotic wave in wild type, without affecting progenitor cell proliferation anterior to the morphogenetic furrow [38](Figure 2C, 2D). In *rux* mutant eye discs, G1 arrest ahead of the furrow was less effective and ended prematurely, with high levels of Cyclin B accumulating in many cells from the earliest stages of R8 specification, and widespread mitosis of R8 and undifferentiated cells from column 2 onwards (Figure 2E, 2F) [28]. GMR-p21 had no discernible effect on any aspect of the cell cycle activity in *rux* mutants (Figure 2G, 2H). This supports the notion that ectopic cell cycles are independent of CycE/Cdk2, although it could also be explained if *rux* mutant cells enter the cell cycle before the GMR-p21 transgene is expressed.

### Mitotic R8 cells do not complete cytokinesis

**Figure 3:**
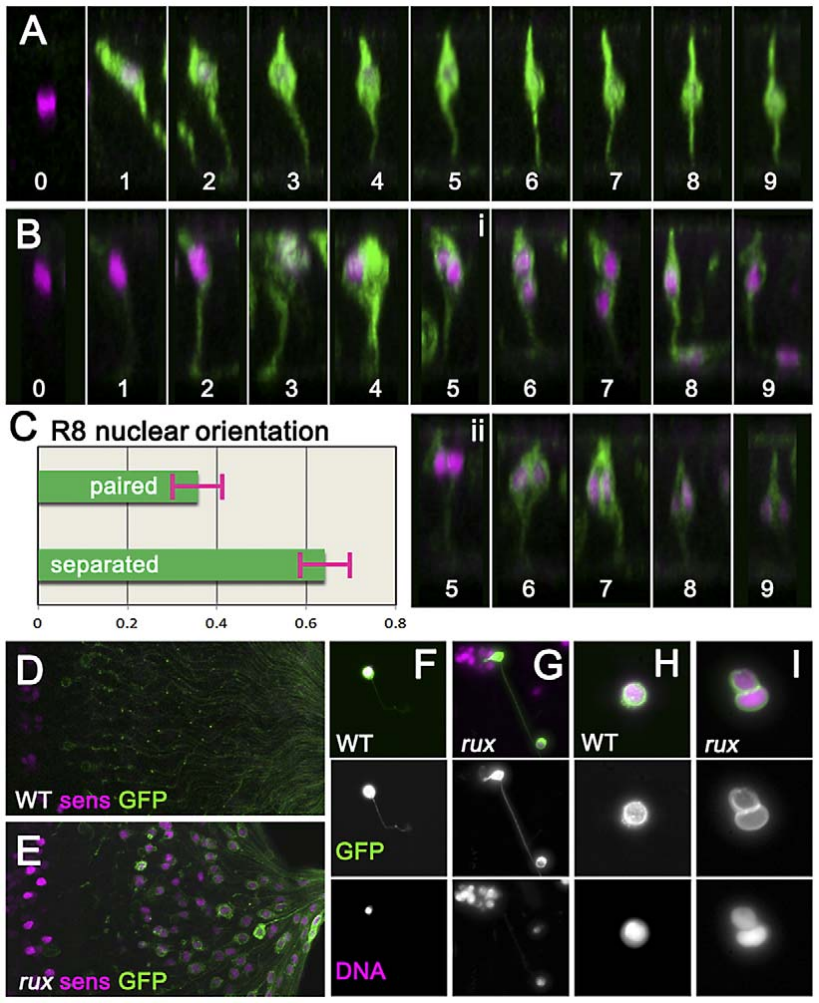
Mitotic R8 cells remain bi-nucleated without completing cytokinesis. (A) Fixed eye discs stained with anti-Sens (magenta). Z-slices of differentiating R8 cells from wild type expressing GFP (green; ommatidial column denoted by numbers). The apical surface is at the top, anterior is to the left. Nuclei rise apically soon after specification, dropping progressively to a somewhat more basal location from column 3 onwards. (B) Z projections of differentiating R8 cells from *rux*. 109-68Gal4>GFP expression is often weaker in *rux*. Around column 3–4, many R8 nuclei are mitotic. Posterior to column 6, most R8 cells retain one nucleus in a normal location while the second drops basally and into the developing axon (i), but in some R8 cells both nuclei remain together in the cell body (ii). (C) quantification of nuclear behavior. (D) the basal third of wild type discs lack R8 nuclei (magenta) near the axons (green). (E) In *rux*, R8 nuclei accumulate in the basal axons. (F) Trypsin treated, dissociated, R8 cells exclusively exhibit a single nucleus as labeled for DNA (magenta). (G) In partially dissociated *rux* mutants, R8 cells containing a second nucleus in the axon can be seen. The figure shows an R8 cell whose cell body remains within an ommatidial cluster dissociated from the rest of the eye disc. (H). After extensive dissociation, wt R8 cells become round without axons. (I) 35% of *rux* 35% cells appear binucleated. See also Video S1.

To characterize the effects of cell cycle re-entry on R8 cells in more detail the G109-68-Gal4 driver was used to express CD8:GFP, a fusion protein effective for labeling neurons [45]. In eye discs, G109-68 expression is almost entirely restricted to R8 cells (Figure 3A) [46]. In confocal micrographs of otherwise wild type eye discs, R8 nuclei ascend apically within the eye disc epithelial layer following specification and then by column 3 drop somewhat as additional photoreceptors are recruited to each ommatidium, but remain in the apical half of the disc epithelium [23](Figure 3A). In *rux* mutant eye discs, R8 morphology resembled normal until column 3 (Figure 3B). By column 6, most *rux* R8 cells contained two separated nuclei within which nuclear proteins such as Sens and Elav appeared confined. Horizontal pairs of nuclei occupied an apico-basal location similar to that of R8 cells from wild type in 38% of such cells (Figure 3C). In most, however, one R8 nucleus at the normal apical position was accompanied by a second progressively more basal (Figure 3B). In columns 15–20, 36.5% of these basal R8 nuclei were found among the axons under the eye disc epithelium, which are normally devoid of nuclei (Figure 3D,3E). These observations suggested R8 cells remained binucleate in *rux*, with one nucleus often moving into the developing axon.

In order to determine directly whether *rux* R8 cells divide, living eye discs were digested with trypsin [47]. Samples of dissociated cells were generated from late third instar and white pre-pupal eye discs whose R8 cells were marked with 109-68>CD8:GFP. No axonal nuclei were ever seen in samples from wild type discs (Figure 3F). When partially dissociated, *rux* eye discs yielded isolated ommatidia containing GFP labeled R8 cells clearly appearing to contain axonal nuclei (Figure 3G). When completely dissociated, single R8 cells rapidly lost their neuronal morphology and rounded up (this process could be observed and recorded under the microscope and often resulted in nuclear fusion: Video S1). Under conditions where all cells were dissociated, all GFP-labelled R8 cells from wild type preparations were mononculeate (Figure 3H). With *rux* mutant discs, ∼35% of GFP positive cells were binucleated (Figure 3I). Thus, many or all R8 cells from *rux* mutant eye discs had not undergone cytokinesis.

### Mitotic defects in R8 neurons

**Figure 4:**
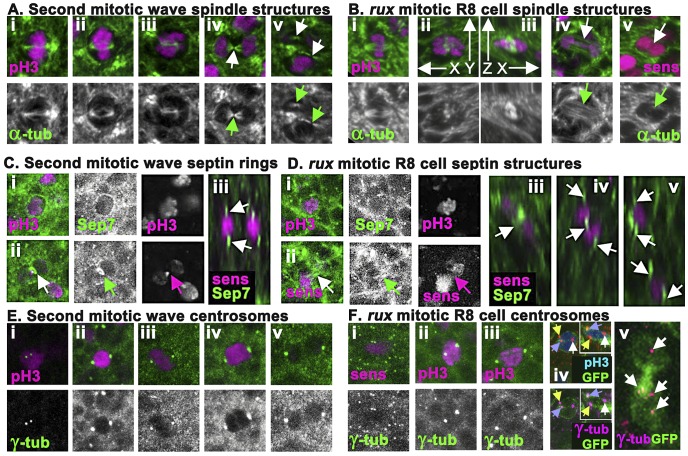
*rux* mutant R8 nuclei separate but exhibit late mitotic defects. (A) Wild type second mitotic wave cells labeled with α-tubulin (green): prophase (i) metaphase (ii) early anaphase (iii), the central spindle (arrow) of a late anaphase cell (iv), and the midbodies indicative of telophase/cytokinesis (arrows) (v). (B) α-tubulin staining (green) of mitotic R8 cells from rux eye discs: a metaphase R8 cell with a spindle parallel to the epithelial surface (i), confocal section through a metaphase R8 cell with a vertically oriented spindle (ii) and a rotated reconstruction of the same cell with apical uppermost (iii). (iv) an R8 cell in anaphase in column 3. Arrow highlights absence of a midbody. (v) an R8 cell in anaphase in column 5. Arrow highlights no apparent central spindle. (C) Septin-7 (green) and H3p or Sens labeling of WT second mitotic waves cells in prophase (i) and telophase (ii). Arrow indicates contractile ring. (iii). An x-y reconstruction of the eye disc showing focal concentrations of Septin-7 apical and basal to photoreceptor nuclei. (D) Septin-7 staining of mitotic R8 cells in prophase (i). Although Septin7 sometmes seems elevated between telophase pronuclei (arrow), no contractile ring is appears (ii). (iii–v) x-y reconstructions of *rux* eye discs showing Septin-7 foci near photoreceptor nuclei, as in wild type. (E) Υ tubulin (green) and H3p (magenta). Prophase cell with recently duplicated centrosomes (i), prometaphase cell (ii), metaphase cell (iii), anaphase cell (iv) and telophase cell (v). Note the background of many tiny foci of Υ tubulin labeling in addition to the centrosomes, thought to represent the location of centrosomes at the apex of G1 cells [56]. (F) Υ tubulin and Sens or H3p (magenta) in R8 cells from rux eye discs: a prophase (i), prometaphase (ii), metaphase (iii). The background of small Υ tubulin foci typical of G1 interphase cells in wild type is replaced in *rux* by apical centrosomes of many dividing cells (i). These are less apparent in panels (ii) and (iii) because R8 nuclei are typically more basally located at these stages. (iv) A subset of mitotic R8 cells appear to contain extra centrosomes. (v) x-z reconstruction confirms that all three centrosomes share one R8 cell. (vi) A binucleated R8 cell in column 7containing multiple Υ tubulin structures (the Υ-tubulin signal has been gain amplified to reveal these weakly-stained structures). See also Figure S2.

To explore how cell division fails in R8 neurons, mitotic figures were examined in neuronal and non-neuronal cells from fixed eye discs from wild type and *rux* mutant discs. Mitotic spindles were characterized using antibodies against α-tubulin. Dividing undifferentiated cells in the SMW from wild type exhibited characteristic prophase, metaphase, anaphase, and telophase morphologies that have been described in other cells, although SMW cells were smaller and embedded in a more complex tissue than S2 cells or embryonic cleavage nuclei [48]–[50](Figure 4Ai–iv). During cytokinesis the nuclear material was entirely separated in each of the daughter cells, H3p diminished, and midbody remnants of the central spindle were observed (Figure 4Av and data not shown).

The spindle morphology of non-neuronal cells dividing in *rux* mutant eye discs could not be distinguished from those of wild type, except that the number of such divisions was greatly increased in *rux* (Figure S2). Mitotic R8 neurons in *rux* exhibited relatively normal spindles through metaphase (Figure 4Bi–iii), but about 20% (n = 50) had spindles oriented perpendicular to the epithelial surface (Figure 4Bii–iii), not parallel as typical of epithelial cells [51], [52]. By anaphase and telophase, however, most R8 cells lacked central spindles (compare Figure 4Biv with Figure 4Aiv). Although R8 pronuclei separated, the midbodies typical of cytokinesis were rarely present (compare Figure 4Av with Figure 4Bv). It may be noteworthy that R8 spindles appeared more normal closer to the morphogenetic furrow. Normally, mitotic cells lose their basal attachments and rise apically in the eye disc so the cells divide within the epithelial plane above the level of most other cells in the disc [14]. In *rux*, mitotic nuclei in R8 cells within the first few columns posterior to the morphogenetic furrow were near the apical surface, but after column 8 H3p labeled chromosome condensed in mitotic R8 cells often remained at their original location more than 5 microns from the apical disc surface (data not shown).

To monitor contractile ring dynamics, we stained third instar eye discs with antibodies against the *Drosophila* homolog of Septin 7 (aka Peanut), which labels contractile ring structures from membrane ingression to the completion of cytokinesis [53]. In undifferentiated cells dividing in the Second Mitotic Wave of wild type eye discs, Septin7 surrounded mitotic cells until anaphase (Figure 4Ci), when it began concentrating to a band around the central spindle that occupied <1 µ between nearly separated cells by the end of cytokinesis (Figure 4Cii). In wildtype, Septin 7 was also evident in differentiating, postmitotic photoreceptor neurons, concentrated above and below the nuclei (Figure 4Ciii).

Septin7 labeling of undifferentiated cells dividing in *rux* mutant eye discs was indistinguishable from wild type (Figure S2). In mitotic R8 cells, Septin7 surrounded the apical cortex (Figure 4Di), but never concentrated into tight bands between separating pronuclei, irrespective of the division axis (Figure 4Dii). After division, Septin 7 concentrated in juxtanuclear foci in the binucleated R8 cells (Figure 4Diii–v). These findings corroborate the conclusion from α-tubulin labeling that mitotic R8 neurons are abnormal by telophase and lack structures typical of cytokinesis.

To characterize the centrosomes we stained Υ-tubulin with antibody GTU-88. Υ-tubulin is associated with pericentric material surrounding centromeres where it acts to nucleate and anchor microtubules to orient and regulate the spindle [54], [55]. This antibody also weakly detailed spindles, and post-mitotic eye disc cells in wild type exhibit a weak apical spot of Υ-tubulin thought to represent the location of the centrosome in G1 cells [56] (Figure 4E). In wild type eye discs, second mitotic wave cells exhibited duplicated centrosomes around column 2; these increased in size and separated to define opposite poles of the mitotic spindle during mitotic prophase, metaphase and anaphase (Figure 4Ei–iv). The centrosomes separated into daughter cells with cytokinesis (Figure 4Ev), then Υ-tubulin labeling faded.

In *rux* discs, centrosome dynamics of non-neuronal cells resembled that of wild type (Figure S2). The centrosomes of mitotic R8 neurons mostly followed a similar mitotic program (Figure 4Fi–iii), with the exception that about 20% appeared to have one or more extra centrosomes based on Υ-tubulin labeling (Figure 4Fiv). Extra centrosomes might result from the ability of Cyclin A/CDK1 to promote centrosome nucleation [57]. They were not associated with multipolar spindles (data not shown). In *rux* R8 neurons after column 7 the weaker Υ-tubulin labeling typical of G1 cells was usually observed just apical to each nucleus, and some cells contained multiple such puncta (Figure 4Fv).

### Kinesin Heavy Chain mediates movement of R8 nuclei

**Figure 5:**
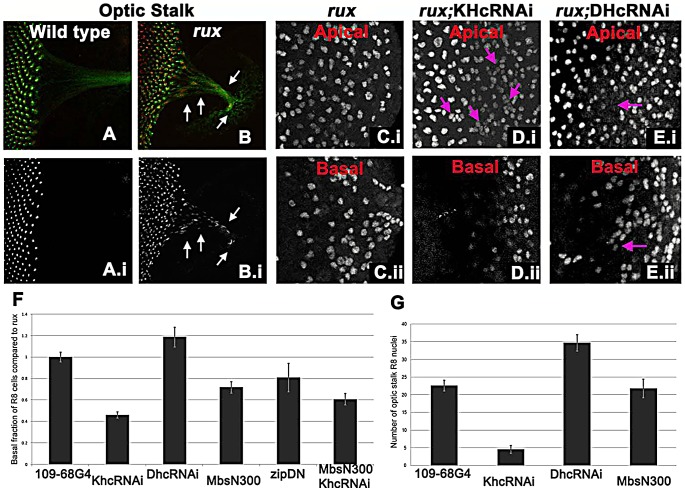
Kinesin and Dynein regulate nuclear position in post mitotic *rux* R8 cells. (A–B) Wild type (A) and *rux* (B) eye discs and optic stalks labeled with anti-Sens (red) and anti-Futsch (mAb22C10; green). Arrows showing R8 cells in the optic stalk in *rux*. (C) R8 cells from the apical (i) and basal (ii) portions of *rux* eye discs expressing GFP. (D) Coexpressing dsRNA for KHC retains R8 nuclei apically (i) while fewer drop basally (ii). Arrows indicate clusters of R8 nuclei apically. (E) Coexpressing dsRNA for DHC leads to apical regions lacking R8 nuclei (arrow in (i) while R8 nuclei accumulate basally (arrow in ii). (F). Results are quantified as the percentage of R8 nuclei observed in the bottom third of the eye disc (in all cases n>2000, from at least 5 different eye discs) and have been normalized to *rux* alone (35%). bars indicate standard errors. (G) The average number of R8 nuclei identified in the optic stalk of *rux* mutants, and discs expressing inhibitors of molecular motors under the control of G109-68-Gal4 (n>5 in all cases, bars indicate standard error).

A striking feature of the *rux* mutant phenotype was the behavior of R8 nuclei after mitosis. In the majority of R8 cells, one nucleus retained a normal position in the cell body while the other traveled basally and into the developing axon (Figure 3B). Accordingly, the number of basal nuclei progressively increased in the posterior region of *rux* eye discs (Figure 3E), and R8 nuclei were even found in the optic stalk, the conduit for retinal axons to the optic lobe (Figure 5A, 5B).

To determine if a motor acts on R8 nuclei, kinesin, dynein, and myosin function were inhibited in *rux* mutant R8 neurons. To investigate the role of kinesin, a plus-end microtubule motor, dsRNA against Kinesin heavy chain (Khc) was expressed in R8 cells using 109-68 Gal4. This had no effect on the morphology of photoreceptors in the wild type background (data not shown). In *rux*, we quantified the proportion of R8 nuclei found in the basal one-third of the eye disc posterior to column 10, including the axons that run along the base of the epithelium (Figure 5C). Khc dsRNA reduced the number of basal R8 nuclei in *rux* by more than half (Figure 5D, 5F). The total number of R8 nuclei was unaffected, resulting in more R8 nuclei clustered at the apical surface (Figure 5D). Knocking down Khc also reduced the number of R8 nuclei that migrate into the optic stalk by over 80% (Figure 5G). Similar results were obtained with two independent dsRNA constructs (data not shown).

Dynein knockdown in R8 cells had no effect on R8 nuclear position in the wild type (data not shown). In *rux* larvae, dsRNAi against Dhc increased the basal R8 nuclei by nearly 20% (Figure 5E, 5F). As some apical disc regions lacked R8 nuclei (Figure 5E) it is likely that both daughter nuclei became basal in some R8 cells. The number of R8 nuclei in the optic stalk increased (Figure 5G), suggesting an increased rate of movement.

We also inhibited Myosin, the primary source of mechanical force in the contractile ring, by expressing *Mbs^N300^*, a constitutively active form of the myosin binding subunit (Mbs) a central component of myosin phosphatase [58]. *Mbs^N300^* expression in R8 cells reduced basal R8 nuclei in *rux* by almost 30% (Figure 5F). A similar result was obtained after expressing dominant-negative Myosin II (*zip^DN^*), but there was no additional effect of doubling the *Mbs^N300^* transgene copy number, or coexpressing *Mbs^N300^* with Khc dsRNA (Figure 5F and data not shown).

Taken together, these results suggest that the nuclei in *rux* R8 neurons are moved into and through axons by kinesin, acting in opposition to dynein. Myosin II may be contribute to all movement, and affect the location of nuclei in *rux* R8 cells indirectly.

### The Anaphase Promoting Complex/Cyclosome also maintains cell cycle exit of R8 cells

**Figure 6:**
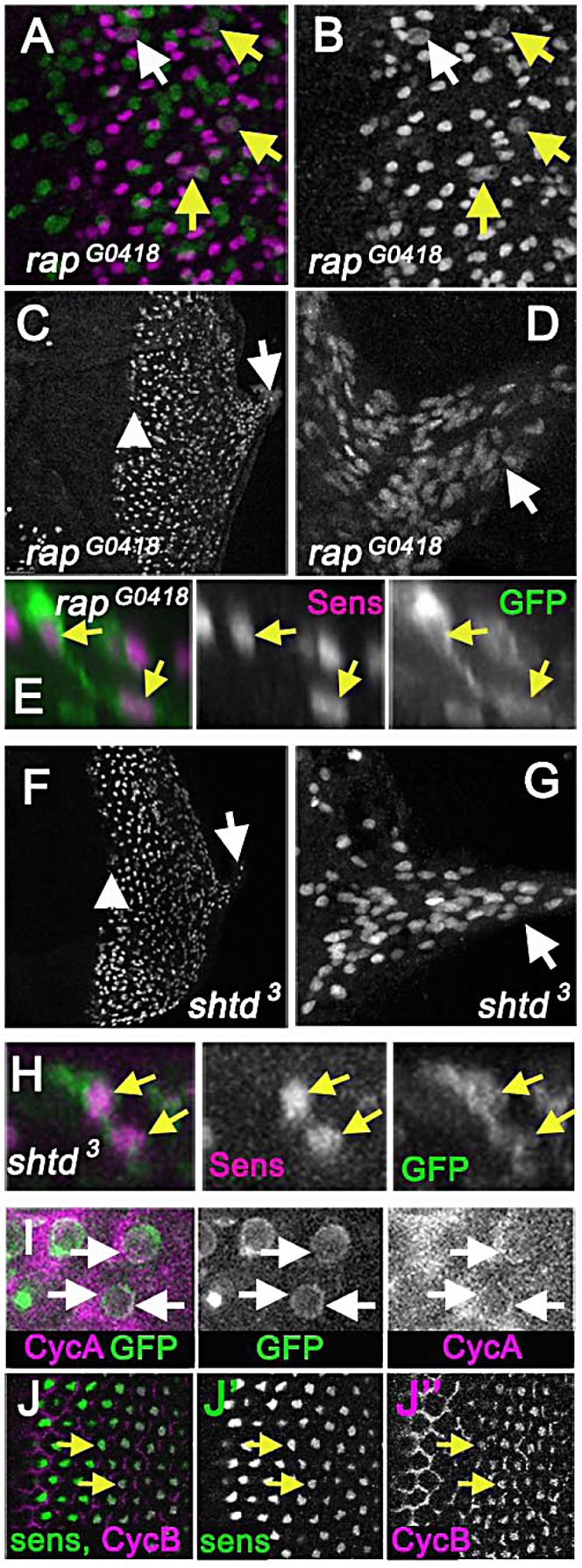
APC1 and Cdh1 maintain cell cycle exit. (A) Eye disc carrying the *rap^G0418^* mutation in the *cdh1* locus labelled for Senseless (magenta) and mitotic figures (anti-H3p in green) showing mitotic R8 cells (arrows). R8 neurons appeared to enter the cell cycle earlier than in *rux*, so that nearly 10% of intermediate groups contained a mitotic cell and 20% of R8 cells in column 0 labeled with H3p (white arrow). (B) Sens channel reveals mitotic R8 nuclei (arrows). (C) R8 cells become disorganized soon after the morphogenetic furrow (arrow head). R8 nuclei can be seen in the optic stalk (arrow). (D) R8 nuclei labeled with sens in the optic stalk of a *rap^G0418^* eye disc. (E) A binucleated *rap^G0418^* mutant R8 cell expressing GFP (green) and labeled for Sens (magenta). (F) Senseless staining of an eye discs carrying the *shtd^3^* mutation in the *apc1* locus. R8 cells become disorganized soon after the morphogenetic furrow (arrow head). R8 nuclei can be seen in the optic stalk (arrow). (G) Enlargement of *shtd^3^* optic stalk. (H) A binucleated *rap^G0418^* mutant R8 cell expressing GFP (green) and labeled for Sens (magenta). (I) Cytoplasmic Cyclin A (magenta) is observed around the R8 nuclei in columns 0 and 1 (arrows), in addition to the labeling of unspecified cells in the second mitotic wave observed previously [28]. R8 cells labeled by 109-68>GFP (green). (J) Cyclin B staining (magenta) co-localizes with senseless (green) staining in wild type eye discs as beginning in column 4, and in all R8 cells by column 6 (arrows). Cyclin B appears nuclear in R8 cells.

Cyclins A and B activities are kept low in most G1 cells by the Anaphase Promoting Complex/Cyclosome (APC/C), a ubiquitin ligase complex that normally remains active until the G1-S transition [59]. Viable hypomorphic mutations of APC/C subunits Cdh1 and APC1 cause rough eye phenotypes [60]–[62]. Mitotic neurons had not previously been described in *cdh1* mutations. Very similar to the situation for *rux*, mutations in *cdh1* (also known as *retina aberrant in pattern*: *rap*) increase cycling of undifferentiated progenitors, and yet mosaic analysis shows that the adult eye defects in *rap* mutants are caused by a requirement in R8 neurons [60], [61]. We report that in eye discs mutant for the *cdh1^G0418^* allele, many R8 cells labeled with the mitotic marker H3p (Figure 6A, 6B). Pairs of apparent postmitotic R8 nuclei were abundant. R8 nuclei moved basally in the eye disc and into axons and the optic stalk (Figure 6C,D) and R8 cells were binucleated (Figure 6E). Thus it appears that just as for *rux*, mutations in *cdh1* affect not only undifferentiated progenitor cells, but also return the R8 neurons to the cell cycle, which is responsible for the major patterning defects. Like *rux* and *rap*, the *shtd^3^* mutant allele of Apc1 exhibits cell cycle re-entry by R8 cells [62]. We find that in *shtd^3^* mutant eye discs, post-mitotic nuclei are also transported basally in the R8 cells and into the axons of the optic stalk (Figure 6F, 6G), and binucleated R8 cells were seen (Figure 6H). Taken together these data indicate that *rux*, *cdh1* and *apc1* are all required to prevent R8 neurons from re-entering the cell cycle, and that cell cycle entry always leads to a similar phenotype of acytokinetic mitosis and nuclear mislocalization in these three mutants.

### Multiple cell cycle proteins are expressed in wild-type R8 neurons

Cyclin A could sometimes be detected in R8 cells in columns 0 and 1 close to the furrow, the stage where R8 cells enter S phase in *rux* mutants (Figure 6I), highlighting the importance of mechanisms to prevent inappropriate cell cycle entry. When eye discs were labeled with antibodies for cdk1 and cdk2, these proteins were seen to be ubiquitous in both neurons and progenitor cells (data not shown). It is already known that Cyclin E accumulates in post-mitotic photoreceptor neuron precursors [3]. Unexpectedly, Cyclin B antibody clearly labels wild type post-mitotic R8 cells after column 5 in the wild type (Figure 6J). Neuronal expression of all these proteins, thought to be specific for the cell cycle, is surprising and emphasizes the importance of post-translational suppression of the cell cycle to these neurons.

## Discussion

Here we show that the *rux* mutation produces a defective *Drosophila* eye because of cell cycle re-entry by differentiating photoreceptor neurons of the R8 class. The *rux* gene encodes a CDI that affects the stability and function of Cyclin A [30], [39]. Cyclin A is normally required in G2/M in *Drosophila*, but if present in G1 can obviate the requirement for Cyclin E/Cdk2 activity to initiate S phase [29], [35]. Although *rux* predominantly affects the eye and male germline, and does not appear to be conserved in vertebrates [63], failure to maintain R8 cell cycle arrest was also seen in hypomorphic mutant alleles of two components of the Anaphase Promoting Complex, APC1 and Cdh1, which are conserved and function similarly in most cell types [59]. APC/C is active in G1 cells until inactivated by Cyclin E/Cdk2 at the G1/S transition, and so also renders Cyclin A unstable in G1 [64]–[66]. These findings support a critical role for eliminating Cyclin A after the terminal mitosis to ensure cell cycle exit by differentiating R8 photoreceptor neurons and by other classes of retinal photoreceptor neuron, which is achieved by APC/C and *rux* together.

The role of APC/C in maintaining cell cycle exit has recently been highlighted in terminally differentiated epidermal cells and non-neuronal cells of the retina [61], [67]. Rux maintains cell cycle exit by non-neuronal cells in the eye also [28]. Mitotic R8 neurons differed from non-neuronal cells of the same genotypes by performing a nuclear mitosis without cytokinesis. There was no evidence that Cyclin A itself prevents cytokinesis, because none of the non-neuronal cells driven into ectopic cell divisions in the *rux* mutant exhibited abnormalities in cell division (Figure S1), and because previous studies have found that stabilizing Cyclin A does not perturb cytokinesis unless the stabilized forms of Cyclin A are over-expressed [36]. We think that failure to execute cytokinesis likely represents an independent barrier to cell division in these neurons, downstream of the G1 exit that is sensitive to Cyclin A.

Perhaps surprisingly, R8 neurons assembled mitotic spindles that appeared to segregate chromosomes relatively normally. Although other abnormalities were noted, the main findings were the absence of proper contractile ring structures and the rarity and disorganization of central spindles and midbodies typical of anaphase and telophase, which might be related. In tissue culture studies of mitosis, treatments that delay cytokinesis at this stage often abort cell division with subsequent development of polyploid cells, similar to the binuclear R8 cells [68]–[70]. We surmise that the differentiation program of neuronal R8 cells precludes normal spindle development and contractile ring formation. This could be related to axon formation, which begins at about the same time R8 cells enter mitosis in the *rux* mutants [23]. Acytokinetic mitosis has also been reported, however, after inactivation of Rb in post-mitotic inner ear hair cells, a cell type that is excitable but lacks an axon [71]. Another feature of mitotic R8 neurons is that they remain anchored to the basal surface of the eye imaginal disc during their abnormal cell cycle (see Figure 3), unlike the normal divisions of unspecified cells that lose their basal footing during cytokinesis [72].

A striking feature of most mitotic R8 cells is the migration of a daughter nucleus into the developing photoreceptor cell axon. We considered the possibility that the cells remain in an extended telophase, transporting one nucleus into the axon as if to the furthest end of the cell in preparation for cytokinesis, but rule this mechanism out for two reasons. In dividing cells the pronuclei are separated by dynein pulling microtubules towards spindle poles, reeling them in towards the centrosome [73]. If such a mechanism were moving nuclei in R8 cells, then knocking down dynein heavy chain would suppress nuclear movement. In contrast to this expectation, we observed more rapid nuclear movement when dynein was knocked down, and in some cells both daughter nuclei were found basally. In addition, centrosomes were generally observed apical to R8 cell nuclei at this stage (Figure 4Biv–v), inconsistent with a role moving nuclei in a telophase-like process. We think it unlikely that Rux protein plays a direct role in locating the nucleus, both because one nucleus is always unaffected in *rux* mutant R8 cells, and because *rux* mutations are not dominant modifiers of *Glued*, a dynactin mutant that affects the position of photoreceptor nuclei ([74] and data not shown) By contrast, the nuclear phenotype was suppressed by reducing kinesin heavy chain expression, suggesting that anterograde transport was the transport mechanism.

Anterograde transport of photoreceptor nuclei has been reported in both *Drosophila* and zebrafish when the dynactin complex is inhibited [74]–[77]. It has been proposed that Dynein and the dynactin complex maintain the position of the nucleus in the photoreceptor cell body, opposing the tendency of kinesins to move the nucleus into the axons [76]. Our observations suggest a more complicated model, since the two nuclei within each cell usually behave differently from one another. We suggest that there may be a single dynactin-dependent berth or niche in the cell body that a second nucleus cannot often share.

Although it is often suggested that cell cycle entry is lethal for neurons, polyploid neurons exist, and It has been suggested that mammalian neurons that re-enter the cell cycle can remain viable by arresting in G2 [4]. In one model, eventual breakdown of the arrest and subsequent entry into M phase is hypothesized as a cause of chronic neuronal death in Alzheimer's Disease [4], [9]. Apparently binucleated neurons resembling mitotic R8 cells in *rux* have been reported in hippocampus from Alzheimer's Disease patients, although rarely [78]. Our findings demonstrate an unexpected connection between failure to exit the cell cycle and axonal trafficking. Defects in axonal trafficking have been implicated in many types of neurodegeneration [10]–[12]. Like *rux*, other mutations in which photoreceptor nuclei are relocated to axons also result in retinal degeneration [74]–[77]. It will now be necessary to determine why mitotic neurons do not form cleavage structures, whether abnormal cytokinesis and nuclear trafficking occur in mitotic neurons of other types, and if such secondary defects of mitotic neurons contribute to neurodegeneration.

## Materials and Methods

### *Drosophila* husbandry and strains

All experiments were conducted at 25 C.

The following fly strains were used in the experiments described above:

y^1^ cv^1^ rux^8^/FM7i, p{ActGFPJMR3} [28]; y^1^ w^*^; P{GawB}sca^109-68^ (Chien et al., 1996); y^1^ w^*^; P{w^+mC^ = UAS-mCD8::GFP.L}LL5 [45]; w; UAS-*MbsN300* [58]; y^1^ v^1^; P{y^+t7.7^ v^+t1.8^ = TRiP.JF01939}attP2 (KhcRNAi) [79];b1 pr1 Khc8/Cyo [80]; w^1118^; y1 w^*^; P{Sep2-GFP.SG}3 [81]; w^67c23^ P{lacW}rap^G0418^/FM7a [82]y^1^ w^1118^ shtd^3^/FM7i, P{ActGFP}JMR3 [62]; P{GD12258}v28053 and w^1118^; P{GD12278}v44338 [37; VDRC]; cycA^C8LR1^.

### Immunohistochemistry

Antibody labeling was conducted as previously described [83]. Antibodies: Guinea pig-anti Sensless [20]; mAb22C10; mouse anti-PNUT (mAb4C9H4) [53]; mouse anti-Cyclin B (mAbF2F4) and mouse anti-Cyclin A (mAbA12) [33]; rabbit anti-phospho-Histone 3 (Upstate Laboratories); rat-anti GFP (Nacalia, USA); mouse-anti-tubulin-α- Ab-2 (Thermo Scientific); mouse anti-Υ-tubulin, Clone GTU-88 (Sigma Aldrich). Secondary antibodies were conjugated to Cy2, Cy3, or Cy5 (Jackson ImmunoResearch Laboratories). Confocal microscopy was conducted using BioRad 2000, Leica SP2 or Leica SP5 instruments. Single channels were collected and analyzed separately in NIH ImageJ1.6 software, and figures were arranged in Photoshop.

### Cell dissociation assays

Eye imaginal discs were dissected and separated from antennal discs in PBS buffer and transferred to 4 ml Trypsin EDTA (Sigma) in PBS, containing Draq5 (diluted 1∶500; Bio Status Limited) in batches of 10–20 and dissociated as previously described [47]. Partial dissociated samples were imaged for 30–45 minutes after 90 minutes of gentle nutation at room temperature, and discs were fully dissociated after 150 minutes of gentle nutation. Epi-fluorescence imaging was carried out using on DeltaVision Core, with an mCherry/GFP filter set.

## Supporting Information

Figure S1 R8 cell mitosis depends on CycA dose. Related to Figure 1. Eye discs labeled for Senseless (magenta; also shown below as separate channel) and phospho-H3 (green). (A) Wild type. (B) *rux^8^*. 60% of R8 nuclei label for H3p by column 4. The number of R8 nuclei has doubled by column 8. (C) *rux* ^8^ *cycA^C8LR1^*/+. 18% of R8 nuclei label for H3p by column 4. The number of R8 nuclei is ∼30% increased by column 8. Thus, R8 mitosis in *rux* depends on CycA gene dose.
(TIF)

Figure S2 Mitosis in unspecified cells in *rux* eye discs. Related to Figure 4. (A) Undifferentiated cells from *rux* eyediscs labeled with Υ tubulin (green) and H3p (magenta). Prophase cell with recently duplicated centrosomes (i), prometaphase cell (ii), metaphase cell (iii), anaphase cell (iv) and telophase cell (v). (B) Undifferentiated cells from *rux* eyediscs labeled with Υ tubulin (green) and H3p (magenta): prophase (i) metaphase (ii) early anaphase (iii), the central spindle (arrow) of a late anaphase cell (iv), and the midbody indicative of telophase/cytokinesis (v). (C) Septin-7 (green) and H3p (magenta) labeling of *rux* second mitotic waves cells in prophase, Arrows indicates contractile ring. Cortical Septin-7 stianing is seen in cells from prophase (i) through metaphase (ii). Contractile rings (arrows) form late in anaphase (iii) and constrict as the cell progresses through telophase (iv) until it forms a small and clearly defined band during cytokinesis (v).
(TIF)

Video S1 Collapse and lysis of binucleated R8 after dissociation. Related to Figure 3. Live imaging of 10968-Gal4>GFP labeled R8 cells stained with DraQ5. Neural cells from trypsin-dissociated *ruc* discs in which nuclei initially appear separated tend to collapse to single round binucleate cell, and may then lyse. Such fusion is further evidence that pairs of nuclei do share the cytoplasm of the same cell. Based on confocal images of fixed tissue, we expected twice as many binucleated cells from *rux* eye discs. Both axon shearing during dissociation and collapse and lysis after dissociation may explain the shortfall.
(AVI)
