# Supplementary figures and images for: Mitosis in Neurons: Roughex and APC/C Maintain Cell Cycle Exit to Prevent Cytokinetic and Axonal Defects in Drosophila Photoreceptor Neurons
Source: PLoS Genet. 2012 Nov 29;8(11):e1003049. doi: 10.1371/journal.pgen.1003049 (PMC3510051; doi:10.1371/journal.pgen.1003049)

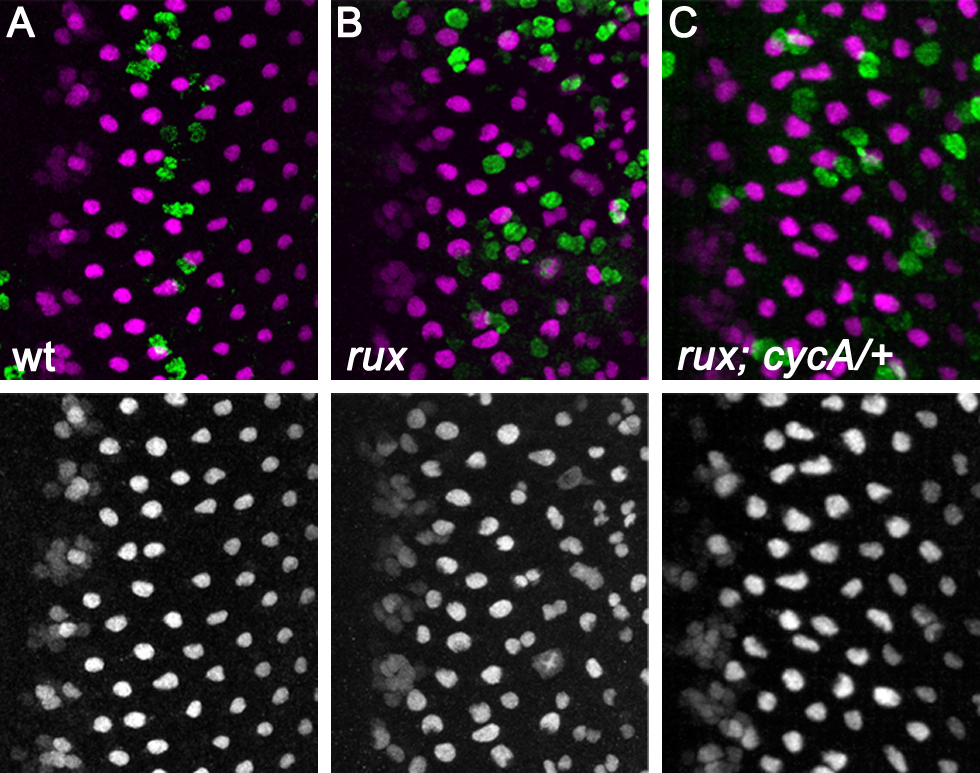

Supplement: Figure S1 — R8 cell mitosis depends on CycA dose. Related to Figure 1. Eye discs labeled for Senseless (magenta; also shown below as separate channel) and phospho-H3 (green). (A) Wild type. (B) rux8. 60% of R8 nuclei label for H3p by column 4. The number of R8 nuclei has doubled by column 8. (C) rux 8 cycAC8LR1/+. 18% of R8 nuclei label for H3p by column 4. The number of R8 nuclei is ∼30% increased by column 8. Thus, R8 mitosis in rux depends on CycA gene dose. (TIF) [file pgen.1003049.s001.tif]

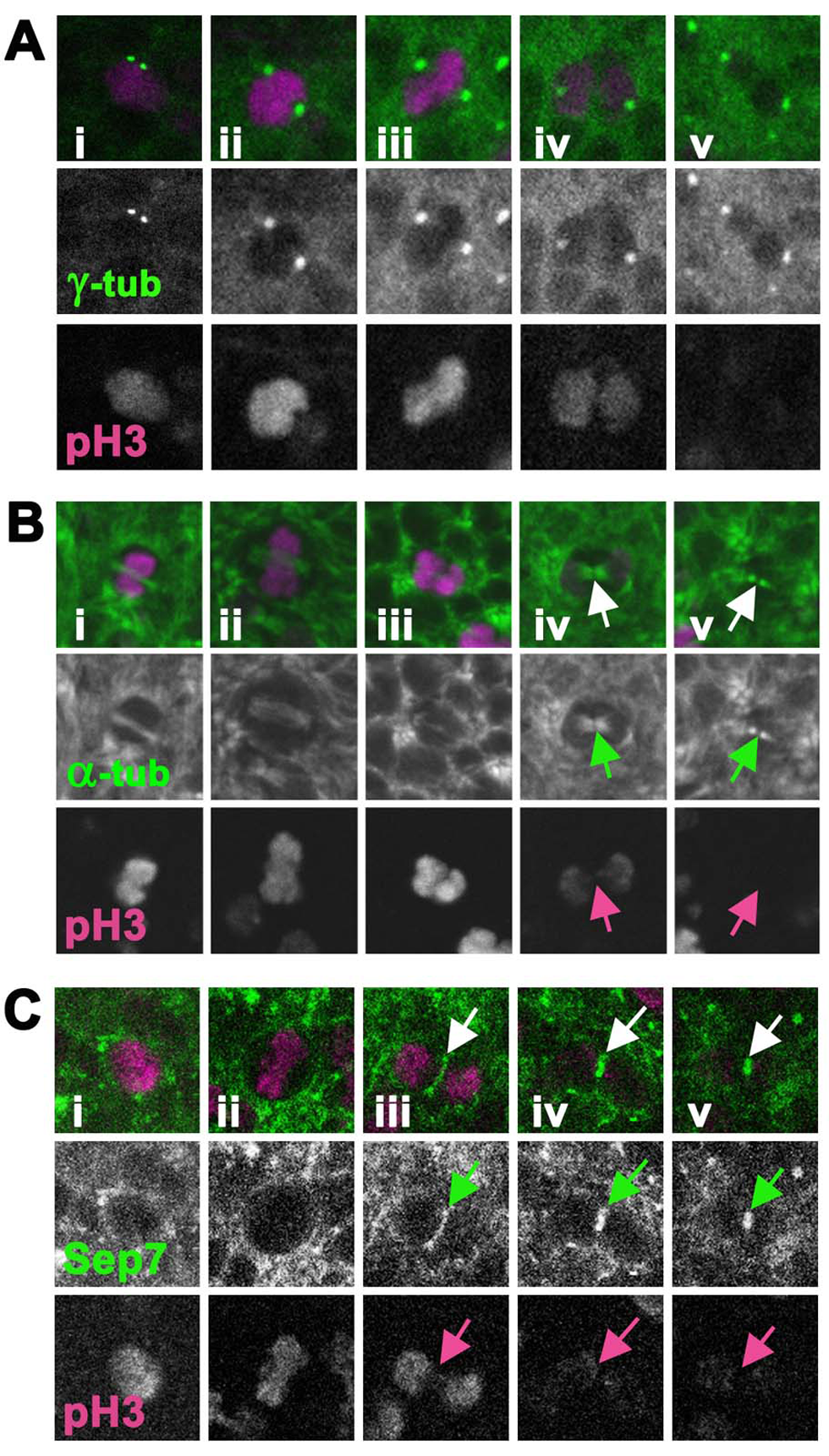

Supplement: Figure S2 — Mitosis in unspecified cells in rux eye discs. Related to Figure 4. (A) Undifferentiated cells from rux eyediscs labeled with Υ tubulin (green) and H3p (magenta). Prophase cell with recently duplicated centrosomes (i), prometaphase cell (ii), metaphase cell (iii), anaphase cell (iv) and telophase cell (v). (B) Undifferentiated cells from rux eyediscs labeled with Υ tubulin (green) and H3p (magenta): prophase (i) metaphase (ii) early anaphase (iii), the central spindle (arrow) of a late anaphase cell (iv), and the midbody indicative of telophase/cytokinesis (v). (C) Septin-7 (green) and H3p (magenta) labeling of rux second mitotic waves cells in prophase, Arrows indicates contractile ring. Cortical Septin-7 stianing is seen in cells from prophase (i) through metaphase (ii). Contractile rings (arrows) form late in anaphase (iii) and constrict as the cell progresses through telophase (iv) until it forms a small and clearly defined band during cytokinesis (v). (TIF) [file pgen.1003049.s002.tif]
